# Unstable aid, unstable lives: a call for reinforced global health solidarity

**DOI:** 10.3389/fpubh.2026.1761358

**Published:** 2026-02-20

**Authors:** Sachchi Baral Chitrakar, Jae Wook Choi, Kyung Hee Kim

**Affiliations:** 1 Center for Global Health Strategy, Institute for Environmental Health, Korea University, Seoul, Republic of Korea; 2 Department of Preventive Medicine, Korea University College of Medicine, Seoul, Republic of Korea; 3 Department of Global Community Health, Graduate School of Public Health, Korea University, Seoul, Republic of Korea; 4 Institute for International Development Cooperation, Korea University, Seoul, Republic of Korea

**Keywords:** aid volatility, DAH, global health, health system resilience, multilateral aid, Sustainable Development Goals (SDGs)

## Abstract

Recent reductions in global health aid, notably the 2025 freeze of USAID funding, have severely disrupted essential health programs in over 130 countries. This disruption has hit essential health interventions such as HIV/AIDS treatment, maternal health, and nutritional support the hardest. Shift in donor engagement along with the sudden USAID freeze brought systemic vulnerabilities in health systems highly reliant on external assistance, particularly across countries with fragile infrastructures. Reduced contributions from major donors, including the United States and United Kingdom, alongside declining multilateral funding, have critically weakened institutions like WHO, UNICEF, Gavi, and the Global Fund. Current projections indicate significant increases in preventable maternal deaths, unintended pregnancies, and HIV infections, threatening to reverse the global health progress of two decades. This perspective analyzes the implications of shifting donor strategies and underscores the urgency of reinforcing multilateral cooperation through Aid Volatility/Shocks Framework to mitigate the cascading effects of acute funding shocks on global health security and preventable mortality. Drawing on recent successes such as COVAX’s global vaccine distribution and the effectiveness of multilateral partnerships during health emergencies, we propose a forward-looking framework: a multi-lateral “anti-shock resilience” model that includes predictable financing, coordinated emergency response, and strengthened governance mechanisms. To achieve these objectives, it requires immediate donor re-engagement, institutional strengthening, and flexible funding models. Ultimately, the ongoing aid crisis highlights the need to prioritize a transition from dependency-driven approaches towards a global health architecture that is sustainable, equitable, and resilient.

## 1 Introduction

The landscape of global health is currently in an unprecedented financial collapse, triggered by the sudden halt and official dissolution of the U.S. Agency for International Development (USAID) and the simultaneous withdrawal from the World Health Organization (WHO) in early 2025. These decisions precipitated widespread disruption across Africa and Asia, where many health system rely heavily on external financing (1). This unilateral funding withdrawal of approximately $9 billion triggered an immediate and systemic 21% contraction of total global Development Assistance for Health (DAH) (2, 3). Essential programs, including HIV treatment for an estimated 2.3 million individuals, experienced abrupt disruption of services. This abrupt disruption is undoing nearly two decades of shared progress, placing the life-saving care of millions at risk and threatening a substantial reversal in global health security (4).

While catastrophic, this immediate funding crisis exposes a deeper, long-standing structural fragility in international development. This perspective draws on the Aid Volatility/Shocks Framework. This framework has been crucial for understanding how persistent financial unpredictability or Chronic Volatility, from generalized ODA budget retrenchments (5) across major European donors (such as the United Kingdom and Germany) made global health systems uniquely vulnerable to the catastrophic, abrupt donor withdrawal, or Acute Shock (6). The definitions and functional mechanisms through which the financing disruptions translate into adverse health outcomes are summarized in Table 1.

**Table 1:** The Aid Volatility/Shocks Framework.

Framework component	Contextual driver	Functional mechanism (impact chain)
Chronic volatility (structural): persistent, long-term financial unpredictability and budget retrenchment that erodes system resilience over time.	Multi-year ODA retrenchments and sustained unpredictability among major donors (e.g., European aid reductions, including the UK GNI decrease to 0.3%).	Budgetary instability ➔ institutional erosion ➔ programmatic weakening ➔ systemic vulnerability.
Acute shock (event): a sudden, high-magnitude funding disruption that triggers the immediate collapse of weakened systems.	Abrupt dissolution of USAID and withdrawal from the WHO in early 2025.	≈$9B funding withdrawal ➔ 21% contraction in global DAH ➔ service termination affecting ≈2.3 million people ➔ mass excess mortality risk.

The repercussions of this aid volatility extend well beyond fiscal shortfalls. The abrupt retrenchment of U.S. assistance has destabilized global health governance, disrupted disease surveillance systems, and jeopardized gains achieved through long-standing initiatives such as the U.S. President’s Emergency Plan for AIDS Relief (PEPFAR). The withdrawal of U.S. leadership has also created critical gaps across humanitarian relief, food security, and education, particularly in fragile states where American contributions have been central to sustaining essential services. Collectively, these disruptions undermine coordinated international responses to emerging health threats and erode the foundations of global health security (7, 8).

We present an urgent appraisal of the converging epidemiological and geopolitical repercussions of the ongoing aid crisis, including projected excess mortality on a massive scale and heightened risks of antimicrobial and antiretroviral drug resistance, and calls for immediate and comprehensive structural reform. Stabilizing global health outcomes will require transitioning from volatile bilateral funding to a resilient multilateral architecture capable of mitigating shocks and sustaining essential services, reinforcing global health security, and preserving progress toward Sustainable Development Goals (SDG) (9).

## 2 Chronic volatility in and the erosion of donor commitment

The withdrawal of US occurred against a backdrop of Chronic Volatility, characterized by stagnating and unpredictable ODA trends. Although, the COVID-19 pandemic briefly brought global health to the forefront of the donor agendas, triggering a surge in Official Development Assistance (ODA) driven by emergency pandemic responses during 2020–2021 (10). However, this momentum was short-lived. As countries pivoted toward domestic recovery, refugee crises, and geopolitical concerns, such as the conflict in Ukraine, long-term investments stagnated. As of 2025; Sweden, Germany, Norway, Luxembourg, and Denmark continue to meet the target of providing 0.7% of their Gross National Income (GNI) in aid (11), underscoring how exceptional the commitment to global health financing has become. This sustained unpredictability is evident in budget retrenchments across major donor nations:

### 2.1 Europe’s aid retrenchment

The United Kingdom substantially reduced its ODA target from 0.7 to 0.3% of its Gross National Income (GNI) (12), leading to major cuts across key multilateral partners like the Global Fund, UNICEF, and UNFPA. Sweden lowered its aid target from 1 to 0.88% of GNI, reallocating much of the remainder to support Ukraine. France’s 2024 development budget was cut by €742 million (13%), and further cuts were announced by Germany, Finland, and Switzerland (13).

### 2.2 Shifting donor dynamics

The multilateral vacuum created by Western aid retrenchment has not been filled by other emerging powers, further destabilizing the global health landscape. For instance, while China has expanded its role as bilateral donor, its engagement is focused on state-to state bilateral aid rather than multilateral mechanisms during this crisis (14). Furthermore, a declining trend in Chinese aid to Southeast Asia (15), also signals a narrowing of its global engagement which contributes to overall regional funding instability.

These shifts in donor priorities have placed an unsustainable strain on the multilateral frameworks, undermining the programmatic integrity essential health interventions, particularly in disease surveillance and maternal-child health. Specifically, the cessation of U. S. contributions in early 2025 removed approximately 15% of the WHO’s total operating budget based on 2024–2025 biennial funding cycle (16, 17). This withdrawal has precipitated a disproportionate 25% budget shortfall within the WHO’s Health Emergencies Program, directly constraining global outbreak response capacities. This environment of chronic volatility fundamentally weakened the global health architecture, leaving it exceptionally sensitive to the massive U. S. Acute Shock that followed (7).

## 3 The acute shock and cascading system collapse

The closure of USAID marked the peak of this structural instability, triggering an Acute Shock that led to the cancellation of roughly 65% of PEPFAR-funded awards and placing an estimated 2.3 million people at immediate risk of losing treatment. This abrupt collapse in financing has produced three rapid and severe consequences (4).

### 3.1 Governance disruptions

The dissolution of USAID as an independent agency, combined with the withdrawal of the United States from the WHO (16), represents a significant policy shift that destabilizes global health governance. The decision to consolidate the remaining USAID global health functions, including Global Health Security activities, into the State Department’s Bureau of Global Health Security and Diplomacy shifts operational authority from a decentralized technical agency to a politically centralized structure. This reorganization, together with a reduced diplomatic workforce now responsible for these functions, weakens disease surveillance capacity and disrupts coordinated responses to emerging health threats. The absence of U. S. leadership in this governance framework jeopardizes health security and could create power vacuums in vulnerable regions (1, 9).

### 3.2 Epidemiological consequences

Conservative projections indicate that continued funding cuts could lead to more than 14.1 million excess all-age deaths by 2030, including over 4.5 million children under five (18). In the near term (by year-end 2025), estimates show an additional 176,000 adults and children could die from HIV and 62,000 additional people could die from tuberculosis (TB). Critically, the sudden interruption of treatment across millions of patients creates a high risk for the mass emergence and spread of drug-resistant pathogens in both HIV and TB (8).

Furthermore, Maternal and Child Health services, which rely on integrated community health worker capacity, have been immediately devastated. Projections suggest 34,000 additional pregnancy-related deaths globally in the first year alone (19). Reports from Kenya and the Democratic Republic of Congo (DRC), highlight stockouts of life-saving HIV, TB, and malaria medications, and preventable obstetric complications (20).

### 3.3 Amplified vulnerability in aid dependent states

The shock was disproportionately amplified in countries exhibiting extreme aid dependency, a direct outcome of chronic volatility. In nations like Afghanistan (341% exposure) and South Sudan (235% exposure) – where exposure is defined as U. S. bilateral global health assistance relative to domestic governmental health expenditure, U. S. bilateral global health assistance was not supplementary but functioned as the primary operating budget for the entire public health infrastructure. For these systems, the financial loss was equivalent to a multi-hundred percent revenue deficit, leading to instantaneous system collapse (21).

The synergistic effect of sustained Official Development Assistance (ODA) retrenchment and an acute USAID financing shock has precipitated a systemic failure in health delivery, extending beyond a fiscal deficit. The sudden loss of funding dismantled the operational backbone for last-mile services, evidenced by mass contract cancellation and the critical withdrawal of local health workers—which critically sustained community care, adherence, and supply chain integrity. This resulting erosion of implementation capacity carries immediate humanitarian ramifications (e.g., escalating malnutrition, imperiling education) across fragile settings and directly threatens political stability by risking institutional collapse and creating exploitable power vacuums. The crisis confirms the extreme fragility of health systems reliant on volatile external aid, demanding urgent, structural reform.

## 4 Building anti-shock resilience: a multilateral reform agenda

The sudden, chaotic collapse of aid delivery underscores the need to address the underlying structural fragilities that allowed this catastrophe to unfold. Without fundamental restructuring, the global health architecture will remain vulnerable to the political cycles of a single dominant donor. This reform agenda proposes a new framework for Anti-Shock Resilience built on predictable multilateralism, rigorous accountability, and prioritized emergency response (5, 7, 9). Table 2 outlines the core pillars and mechanisms of this proposed Anti-Shock Resilience framework.

**Table 2:** Proposed structural components of anti-shock resilience.

Strategic pillar	Core objective	Key mechanism and agent	Targeted outcome
Pillar I: immediate emergency response	Restore critical services disrupted by funding shocks	Agile multilateral partnerships (Gavi, Global Fund, UNICEF, WFP, CEPI) leveraging COVID-19 pooled response models (e.g., COVAX)	Sustained ART continuity; restoration of childhood immunization; immediate reduction in excess maternal/child mortality.
	Target extreme geographic vulnerability collapse	Priority-based targeting (2025–2027) focused on Africa and fiscally exposed states; deployment of short-term delivery platforms (mobile clinics, telehealth support)	Immediate stabilization of essential service delivery in the most vulnerable regions (e.g., Afghanistan, South Sudan)
Pillar II: structural anti-shock financing	Achieve fiscal stabilization post-shock	Multi-donor trust funds established by MDBs (World Bank, ADB) pooling reallocated ODA, utilizing burden-sharing formulas	Bridging grants ensuring health worker payroll continuity; uninterrupted procurement of essential, life-saving commodities
	Establish predictable, resilient funding	Multilateral–bilateral hybrid mechanisms, scaled public–private partnerships (PPPs), and development impact bonds (DIBs).	Reduced long-term dependence on politically contingent bilateral aid; mitigation of future chronic volatility

### 4.1 Prioritizing emergency response

In the wake of the acute funding shock, the immediate priority must be “putting out the urgent fires” which means, deploying prioritized, sector-specific interventions to the most affected regions. While critics cite potential bureaucratic inefficiencies within multilateral institutions, the current crisis confirms that these issues are secondary to the existential threat posed by bilateral volatility, which leads to immediate and catastrophic service failure. Multilateral cooperation offers a sustainable and effective alternative to politically contingent bilateral ODA, which often leads to redundancy and fragmentation in program delivery. Therefore, emergency response efforts must be reallocated to institutions with proven operational agility (9).

Multilateral partnerships, such as Gavi, the Global Fund, UNICEF, WFP, and CEPI, have demonstrated the ability to rapidly deliver services, manage pooled financing, and operate across fragile contexts. This agility is critical for restoring services like HIV/AIDS treatment (ensuring ART continuity), childhood vaccination, maternal health support, and therapeutic feeding programs for acute child malnutrition. The feasibility of rapid, large-scale multilateral intervention is evidenced by the COVAX platform, which successfully delivered nearly 2 billion COVID-19 vaccine doses across 146 countries, even under constrained circumstances. (4–7)

Targeted emergency programs must prioritize the continuity and restoration of critical disrupted services, including HIV/AIDS treatment (ensuring ART continuity), childhood vaccination, maternal health support (safe deliveries), and scaling up therapeutic feeding programs for acute child malnutrition.

### 4.2 The anti-shock financing architecture

To stabilize exposed countries, Multilateral institutions led by the World Bank, ADB, and other MDBs, should establish multi-donor trust funds that pool diverse resources and provide bridging grants for sustaining payrolls, procurement systems, and critical health services. These funds must prioritize sustaining health worker payrolls and maintaining uninterrupted procurement of essential, life-saving commodities. At the same time, continuity of existing multi-year commitments in emergency response, immunization, and epidemic control must be ensured, as premature termination risks catastrophic reversals in health gains and exacerbates mortality in already fragile systems.

### 4.3 Leveraging multilateral-bilateral hybrid mechanisms

The effectiveness of multilateral partnerships hinges on predictable funding and structural reforms that strengthen their responsiveness and resilience. Donor nations should increasingly channel resources through multilateral–bilateral hybrid mechanisms that foster coordinated engagement across public, private, and subsidy-based programs, reducing dependence on politically contingent bilateral aid. Expanding collaboration with the private sector is equally vital: public–private partnerships (PPPs) must be scaled to mobilize innovative financing and enhance delivery capacity, drawing on models such as the Global Fund’s successful engagement of over $1 billion in private sector contributions (22). Moreover, innovative mechanisms like Development Impact Bonds (DIBs) could serve as a supplementary tool to align private investment incentives with measurable, outcome-based public health achievements, thereby reinforcing both efficiency and accountability in health financing (23).

## 5 Conclusion (call for action)

Clear priorities and strengthened multilateral cooperation are essential amid escalating disruptions in global health financing. Multilateral global health partnerships such as Gavi, the Global Fund, CEPI, UNICEF, and WFP are well positioned to restore essential services and reinforce global health security, provided they receive flexible and sustained support that reduces dependence on politically variable bilateral funding. Moreover, countries that have maintained strong global health commitments including Korea, Japan, and China, can play a strategic leadership role by reinforcing pooled financing mechanisms and advancing reforms that enhance multilateral responsiveness and resilience. The immediate priority must be the execution of proposed strategic approaches to stabilize vulnerable health systems, close persistent financing gaps, and build a more predictable and equitable global health architecture.

## Data Availability

The original contributions presented in the study are included in the article/supplementary material, further inquiries can be directed to the corresponding author/s.
